# Annotations

**Published:** 1903-08-08

**Authors:** 


### Annotations.

### The Nauheimer Baths.

As a continental health resort Bad-Nauheim is world-renowned. The efficacy of its springs has attracted invalids from all countries, and cases of heart disease particularly are known to do well under the *régime* adopted there. But it is not to be supposed that Nauheim will suit all such cases, nor should it be inferred if Nauheim fails to cure some that the baths are therefore useless and dangerous to others. The physicians of Bad-Nauheim, justly enough, insist that the baths should be taken neither too early nor too often; that the benefit in heart cases can only become evident *peu à peu*. In many instances, after the exertion of travel, treatment preliminary to the use of the baths may be necessary for a considerable time before the stimulating influence of the waters can be commenced. Hence, in case of need, patients should be prepared to stay weeks, or months, if they are to derive lasting benefit. While, with careful and prolonged treatment, successful results may be expected in the cardiac neuroses, influenza-heart, and the slighter forms of endocarditis, the cure of chronic valvular disease, especially when there is already a probability of calcareous degeneration, is of course not possible. Often enough in such cases as the latter, any material benefit derived from the treatment is lost by the fatigue of a long journey. If only just the kind of case suitable went to these health resorts, no disappointment need arise, and patients properly instructed beforehand, in the nature of the treatment, and the probable length of time necessary to effect complete recovery, Nauheim might prove a haven of safety to the majority of its visitors.

### Quacks and Anti-quacks.

The quack who writes a little book to expose the ways of other quacks, is not, as might be supposed, a pervert from quackery, but a firm believer in it, only he practises without drugs; he does not procure testimonials from ignorant persons, and he does not advertise in the religious and lay press. On the contrary, he denounces all these things. With his identity, and his particular hygienic fad, we have nothing to do; but he is a phenomenon of the time, which is worth taking account of. In Germany they have had this new quackery, or anti-quackery, for a long time, so that it is now reduced to a system and united in a kind of social league against the regular faculty. The illustrious Virchow, in one of his last public efforts, thought it incumbent upon him as the leader of scientific medicine, to notice the widespread German revolt from the medicine of the schools, which goes under the name of "Natur-Medicin." It was not necessary for him to enlarge upon the enormous prevalence of quackery proper, for of that the profession in Berlin and elsewhere are only too well aware. Virchow was not content to raise his voice, he did what he could to counteract the evil in a practical way which was characteristic of the man. Believing that nature-medicine was rooted in ignorance, but amenable to instruction, he had a section of the pathological museum laid out with telling specimens of all the morbid processes, so that the public might come and see for themselves what kind of grim thing disease really was. It was certainly a courageous attempt to bridge over the gulf between the highly-equipped and incessantly busy laboratories, clinics, and professorships on the one side, and the popular indifference to their "output" on the other. Whether it is likely to have any effect we do not know. But in Germany—"sapient Germany," as Wordsworth called it in Napoleon's time—we have an object-lesson of the difficulty of getting the people to keep up with the thinkers and researchers. The body-politic appears to be deficient in the function of assimilation. Hence arises a state of plethora in which peccant humours are thrown out upon the surface. The old-fashioned quackery of pills and ointments, syrups and elixirs, flourishes amazingly all round the walls of the scientific institutes, and its active rival is, not the medicine of the schools, but a new quackery which abjures drugs on the one hand and pathology on the other.

### The Insanitary Dustbin.

It must be reckoned as a mystery that in a generation in which so much is said about sanitation, the dustbin survives as a domestic adjunct. Happily, it is not universal. There are country towns, and even villages, where the dust and other refuse is cleared away daily, but in some of the London suburbs, which arrogate to themselves the title of boroughs, and have all the paraphernalia of municipal management—including sanitary officers—the household refuse is allowed to accumulate for a week at a time. Once a week only does the dustcart come round, and it is not to be wondered at that in the circumstances its advent is marked by a malodorousness which taints all the surrounding air. It is true that householders are advised to burn all animal and vegetable matter which is liable to decay. But this is a counsel of perfection. Many people, even among the poor, use gas stoves in the summer-time, in order to keep the house cool, and because, when no general heating is necessary and a fire is required only for cooking, it is more economical. They will not light a fire for the mere sake of burning up refuse, which, moreover, smells not too pleasantly in the burning. Thus at the very season when it is most necessary to get rid of rubbish, it is most certain to be allowed to lie until the authorities remove it. Knowing this, are the municipal authorities justified in allowing it to lie so long? The mass of decomposing matter accumulating in a tiny yard must at any time, but especially in hot weather, have an injurious effect on the health of those who inhale the gases arising from it. Even where it is not possible to trace actual cases of disease to this cause, it may be responsible for a generally lowered vitality, which makes people more prone to catch any ailment that may be about. Surely this is a matter to which public health authorities might give more attention than they do. To clear away rubbish every day might involve more expense in the matter of carts and dustmen; but if, as we believe, the matter really affects the health of the people, it would be a justifiable expense, and might in the end be counterbalanced by such a decrease in preventable disease as would permit of economy in other ways. All the efforts of sanitary authorities are devoted to that prevention, the value of which is proverbial. The daily clearing away of household refuse would surely be a step in that direction

